# Preoperative risk assessment of endometrial cancer using histogram analysis of weighted and quantitative MRI images

**DOI:** 10.1007/s00261-025-05069-6

**Published:** 2025-06-27

**Authors:** Magnus Palmér, Åsa Åkesson, Maria Ljungberg, Stefan Kuczera, Emilia Gryska, Erica de Coursey, Rolf A. Heckemann, Pernilla Dahm Kähler, Stephan E. Maier, Henrik Leonhardt

**Affiliations:** 1 University of Gothenburg, Gothenburg, Sweden; 2 Sahlgrenska University Hospital, Gothenburg, Sweden; 3 Brigham and Women’s Hospital, Boston, United States

**Keywords:** Endometrial cancer, Magnetic resonance imaging, Diffusion MRI, Perfusion MRI

## Abstract

**Objective:**

The aim of this study was to evaluate the capability of histogram analysis of weighted and quantitative MRI images to improve preoperative endometrial cancer (EC) risk stratification by providing information about the histological properties of the tumours.

**Methods:**

In this prospective study, 94 patients with biopsy verified endometrial carcinoma underwent a preoperative MRI examination performed according to the European Society of Urogenital Radiology (ESUR) guidelines with addition of synthetic MRI, dynamic contrast enhancement and diffusion weighted imaging (DWI) with high b-values. Quantitative relaxation maps, perfusion maps and diffusion kurtosis imaging (DKI) maps were generated from the additional sequences. Tumours were segmented on three adjacent slices and histogram properties were compared between tumours with low and high histological risk.

**Results:**

Significant differences were found between tumours with low and high histological risk in the histogram properties for the DKI derived apparent diffusion maps (D_app_): mean (p = 0.048), median (p = 0.025), skewness (p < 0.001) and kurtosis (p = 0.003). No significant differences between the groups were observed in histogram properties of quantitative relaxation maps, acquired by synthetic MRI.

**Conclusion:**

Histogram analysis of DKI shows better potential to discriminate between EC histological risk groups and histologically determined endometrioid tumour grades than regular DWI, relaxation maps from synthetic MR, perfusion maps and T1 or T2 weighted images.

## Introduction

Endometrial cancer (EC) is the most common gynecological malignancy. Most endometrial tumours have an excellent prognosis after standard treatment with hysterectomy and bilateral salpingo-oophorectomy. The five-year relative survival rate is almost 85% [1, 2]. However, some tumours have an increased risk of metastatic disease, mainly lymph node metastases, which is associated with a worse prognosis. For endometrial tumours with a higher risk of metastases, surgical lymph node assessment is recommended for staging to tailor adjuvant therapy. The extended surgical procedure with lymphadenectomy is more complex and increases the risk of adverse events, mainly lymphedema [3, 4].

Preoperative risk assessment may be performed to stratify patients into preoperative low-risk or high-risk groups based on a combination of histological findings at biopsy and the local tumour extent seen on imaging, i.e., mainly the depth of myometrial invasion. Magnetic resonance imaging (MRI) is recommended by the European Society of Urogenital Radiology (ESUR) as the primary imaging modality. However, the reported diagnostic accuracy in estimating myometrial depth invasion varies [5–8].

The endometrioid differentiation is graded, according to the International Federation of Gynecology and Obstetrics (FIGO), into three grades, i.e., FIGO grade 1, 2, or 3 (G1, G2, or G3) [9]. G3 endometrioid adenocarcinomas and non-endometrioid tumours are considered high-risk, whereas G1 and G2 endometrioid tumours are generally regarded as low-risk. However, discrepancies between the preoperative biopsy and the final postoperative histopathological assessment occur in approximately 20% of the cases [10–13]. Underestimation of the malignancy grade often necessitates additional surgery, whereas overestimation usually implies that the surgery has been unnecessarily extensive. Improving preoperative tumour risk estimation using MRI by adding information about histology might help avoid surgical overtreatment and improve surgical targeting.

Imaging studies have shown visual differences between tumours of distinct histopathological types and FIGO-grades. As described by Epstein et al. [14], examinations using transvaginal ultrasonography (TVUS) show differences in homogeneity and vascularization between endometrioid tumours of different FIGO-grades.

MRI diffusion weighted imaging (DWI) is recommended in the European Society of Urogenital Radiology (ESUR) guidelines for female pelvic MRI [5]. Apparent diffusion coefficient (ADC) values calculated from diffusion weighted imaging (DWI) are used to quantify the rate of diffusion. In some studies ADC values seem to correlate with EC tumour grade [15–17] and to have the potential to differentiate malignant from benign lesions [16, 18, 19], while other studies were unable to find such a correlation [20–22]. ADC values may also differ between tumours of different growth patterns [15, 23, 24]. Computation of ADC from DWI is based on the assumption that displacement of molecular water follows a Gaussian distribution. This is not given for biological tissues because of the complex microstructure. Diffusion kurtosis imaging (DKI) is an extension of DWI that takes into consideration how much the measured diffusion differs from a hypothetical Gaussian distribution. DKI has been proposed for more comprehensive characterization of water diffusion and tissue structure [25, 26]. In one study, DKI has been reported to be useful in EC tumour grading, but its authors recommended further validation [27].

Synthetic MRI is a quantitative imaging method where the T1 and T2 relaxation times as well as the proton density (PD) are measured in one single short-duration scan [28]. In a prior study on synthetic MRI in EC, no significant differences in T1, T2 or PD values were seen between FIGO grade 1 and FIGO grade 2–3 tumours [15].

The guidelines from ESUR on MRI staging of EC recommend the inclusion of either a dynamic contrast medium enhanced (DCE) MRI or a single 150 s post-injection sequence [5]. Calculating perfusion values from the dynamic contrast enhanced images makes it possible to assess the blood flow and vascularity in a tissue. Previous studies have described differences in perfusion between endometrioid (low-risk) and non-endometrioid uterine (high-risk) malignancies as well as between tumours of distinct FIGO grades [29–32].

Although the correlation between histogram properties, derived from MRI sequences, and EC tumour grade have been studied previously, the results are ambiguous. No study has compared the discriminating capability of histogram analyses of multiple MRI sequences in the same study population. The value of synthetic MRI and DKI are not well investigated in EC.

The primary aim of this study was to enhance the accuracy of preoperative risk stratification in EC. By identifying differences in MRI image properties between tumours with low and high-risk histological features, we aimed to utilize preoperative MRI to provide information on tumour type and grade in addition to the macroscopic growth pattern, ultimately leading to a more accurate preoperative risk stratification.

## Materials and methods

### Recruitment of participants

This single-centre study is part of the larger multi-centre PODEC study (PreOperative Diagnostics of Endometrial Cancer) conducted in the Western Sweden health care region [8]. The study was approved by the Regional Ethical Review Board in Gothenburg (D-no: 527-16), and written informed consent was collected from all patients.

Eligible for inclusion were patients admitted to Sahlgrenska University Hospital with biopsy-verified EC without apparent metastatic disease. All included patients underwent an MRI scan at the university hospital between January 2017 and December 2019. Exclusion criteria were contraindications for MRI, pregnancy, severe kidney disease, and age less than 18 years. Patients with tumours with an anteroposterior extent of less than 10 mm (as measured by at least one radiologist) were also excluded, since tumour segmentation was assumed infeasible on smaller volumes.

### MRI acquisition and image post processing

All examinations were performed on a single 3 Tesla scanner (Philips Achieva dStream, Philips Healthcare, The Netherlands) using Philips posterior and anterior coil, with a maximum of 32 channels. The protocol was designed according to the recommendations by ESUR 2018 [5] and included T1W sequences, T2W sequences sagittal, axial, and axial-oblique perpendicular to the uterine cavity, clinical DWI and DCE sequences axial oblique. In addition, quantitative relaxation maps based on a turbo spin echo (TSE) pulse sequence synthetic MRI (SyMRI, SyntheticMR AB, Linköping, Sweden), and a DWI sequences with 15 distinct b-values evenly distributed in 250 s/mm^2^ increments from b = 0 s/mm^2^ to ultrahigh b = 3500 s/mm^2^ were obtained. The DCE sequences were obtained with 20 samples, one every 11.5 s after injection of Gadolinium-based contrast medium (Gd). Details are listed in Table 1.

**Table 1:** MRI protocol with conventional sequences, Synthetic MRI, diffusion kurtosis imaging and dynamic contrast enhancement

Sequence	Technique	Orientation	FOV (mm)	Acq voxel (mm)	Slice thickness (mm)	Gap (mm)	TE (ms)*	TR (ms)*	Acq time (mm:ss)
T2W	TSE	sag	240 × 271	0.6 × 0.83	3	0.3 mm	100	1700–5000 (~ 4355)	03:46
T2W	TSE	cor	240 × 240	0.6 × 0.95	3	1 mm	100	1700–5000 (~ 4427)	02:04
T2W	TSE	tra	240 × 240	0.6 × 0.95	5	0.5 mm	100	1700–5000 (~ 4427)	02:04
T2W	TSE	tra-obl	200 × 200	0.6 × 0.95	2.5	1 mm	100	1700–5000 (~ 4427)	04:08
DWI 5b	EPI ssh	tra-obl	240 × 240	3.0 × 3.0	4	0.4 mm	98	~ 6370	04:21
DWI 5b	EPI ssh	sag-obl	240 × 240	3.0 × 3.0	4	0.4 mm	98	~ 4251	03:45
Synthetic MRI	TSE	tra-obl	230 × 191	1.1 × 1.1	4.5	0.5 mm	~ 13/100	~ 3051	05:20
T1W dixon-IW	3D FFE	tra-obl	260 × 260	1.6 × 1.5	3	Overlapping 50%	~ 1.33/2.3	~ 3.7	00:39
DWI_15b 3500	EPI ssh	tra-obl	280 × 224	3.6 × 3.6	4	0.4 mm	~ 102	~ 5077	03:45
T1_alfa_5	3D FFE	tra-obl	300 × 258	1.5 × 1.5	3	Overlapping 50%	~ 1.62	~ 3.4	00:21
T1_alfa_15	3D FFE	tra-obl	300 × 258	1.5 × 1.5	3		~ 1.62	~ 3.4	00:27
e-THRIVE/GD	3D FFE	tra-obl	300 × 258	1.5 × 1.5	3		~ 1.55	~ 3.2	03:53
T1W dixon-W	3D FFE	sag-obl	260 × 260	1.6 × 1.5	3		~ 1.43 / 2.3	~ 3.6	00:27

*e-THRIVE*   enhanced T1 high resolution isotropic volume examination, *GD*   gadolinium, *TSE*   turbo spin echo, *EPI*   echo planar imaging, *ssh*   single shot, *3D FFE*   3D fast field echo, *sag*   sagittal, *cor*   coronal, *tra*   transversal, *obl*   oblique, *Acq*   acquisition, *TE*   echo time, TR = repetition time

*For scans where TE and TR is set to shortest, typical values are given. Indicated with ~

From perfusion sequences, the following maps were generated: area under the curve (AUC), the transfer constant (K^trans^) for Gd from plasma to the extravascular extracellular space (EES), the time constant for reflux from Gd from the EES (k_ep_), and the fractional plasma volume contributing to the intravascular part of the signal (v_p_). Maps were created using Intellispace software (Philips Medical Systems, Best, The Netherlands).

From the clinically used DWI sequence, regular mono-exponential ADC maps were calculated using diffusion-weighting values of b = 100 s/mm^2^ and b = 1000 s/mm^2^. For the research DWI sequence with 15 b-values, the apparent diffusional kurtosis (K_app_) and the diffusion coefficient corrected for observed non-Gaussian behaviour (D_app_) were calculated by means of non-linear regression using diffusion-weighting values in the range from b = 250 s/mm^2^ to b = 2750 s/mm^2^, i.e., b-values ≥ b = 3000 s/mm^2^ were not included since the DKI-model results in inferior fits for higher b-values [25, 33].

### Segmentation and feature extraction

The segmentation was manually performed by a radiologist (M.P.) with initially 4 years of experience in gynaecological MRI using 3D Slicer software (Version 4.13.0, Brigham and Women’s Hospital and Massachusetts General Hospital, Boston, MA. Retrieved from https://www.slicer.org) [34]. For each separate MRI sequence, the tumour was delineated on three neighbouring slices.

**Fig. 1:**
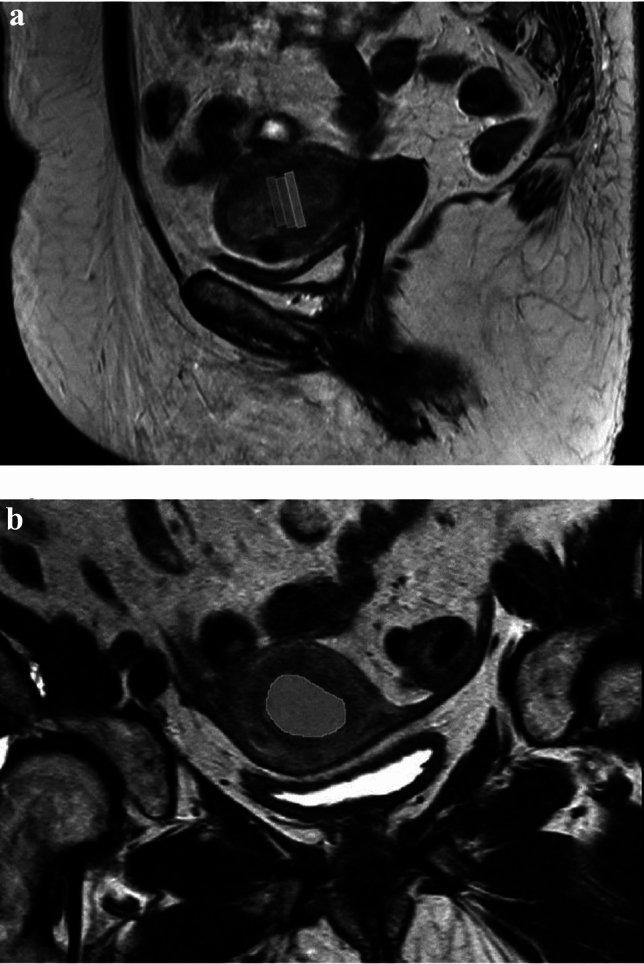
Segmentation on three neighbouring slices. **a** Sagittal T2-weighted image **b** Oblique T2-weighted image axial to the uterus

All tumours were manually delineated on all available MRI sequences on 2D sections, positioned axially with regard to the uterus on the slice with the largest visible area of the tumour, and then on the two neighbouring slices. The slice thickness varied (for T2W 3.5 mm, for T1W including DCE 1.5 mm, for DWI including DKI 4.4 mm and for relaxation maps 5 mm). Due to the differences in pixel size, the number of included pixels from a tumour varied between the MRI sequences. Apparent necrosis and normal endometrium were avoided, in order to include only viable tumour tissue. Figure 1 illustrates the neighbouring slices.

To achieve optimal data extraction, segmentations were outlined separately for each of the included MRI-sequences. For DKI, the segments were drawn on the image with the b-value showing the highest visual contrast between tumour and myometrium. For the relaxation time maps, the segments were drawn on the T2W maps. For DCE, the segments were drawn on the image showing the highest visual contrast between tumour and myometrium.

From the segmentations, the histogram properties were extracted using PyRadiomics version 3.0.1 through the 3D Slicer plug-in [35]. For the non-quantitative sequences, we compared the skewness and kurtosis of the histograms between the group with low histological risk (EC FIGO G1-G2) and the group with high histological risk (EC FIGO G3 and non-endometroid). For the quantitative sequences (ADC-maps, D_app_-maps, relaxation time maps, DCE perfusion), we additionally compared mean and median values. No normalization or histogram-based pre-processing was conducted before analysis.

In a sub-analysis we compared histogram properties from D_app_ and ADC maps between different FIGO grades of EC.

### Radiological evaluation

The tumours were visually assessed by a radiologist (H.L.) with more than 20 years of experience in gynaecological MRI. The purpose of this assessment was to compare the discriminating performance of an experienced radiologist to that of histogram analysis. Assessments were made on the MRI sequences recommended by ESUR, as other maps were generated later in the process. The radiologist classified each tumour as either “low risk histology” or “high risk histology”. The classification was based on the readers experience and overall impression of the tumour. For example, the presence of a single supporting vessel was taken as a sign of low histologic risk as previously shown in an ultrasound study [14]. To our knowledge, there are no similar reports on visual risk evaluation of EC characteristics for MRI.

### Histopathological analysis

The histopathology of the tumour was decided based on the surgical specimen. The tumours were evaluated by reference pathologists, specialized in gynecological cancer.

### Statistical analysis

A significance threshold of 0.05 was used. For comparison of histogram properties between the two histological risk groups, two-tailed independent samples t-tests were used for normally distributed data, either Student’s t-test or Welsh’s t-test depending on variances. The distribution was tested using Shapiro–Wilk’s test of normality. Non-parametric Mann–Whitney-U tests were used for data not normally distributed.

The skewness and kurtosis on the histograms were compared for the non-quantitative values. For quantitative values, the mean and median were compared. Sensitivity and specificity were calculated for the radiologist, and an ROC analysis was carried out. Differences between EC grades in D_app_ and ADC histograms were compared using one-way ANOVA.

The software used was SPSS (SPSS Statistics version 28.0.1.1 for Windows, IBM, Armonk, New York, USA).

## Results

### Clinical characteristics

**Fig. 2:**
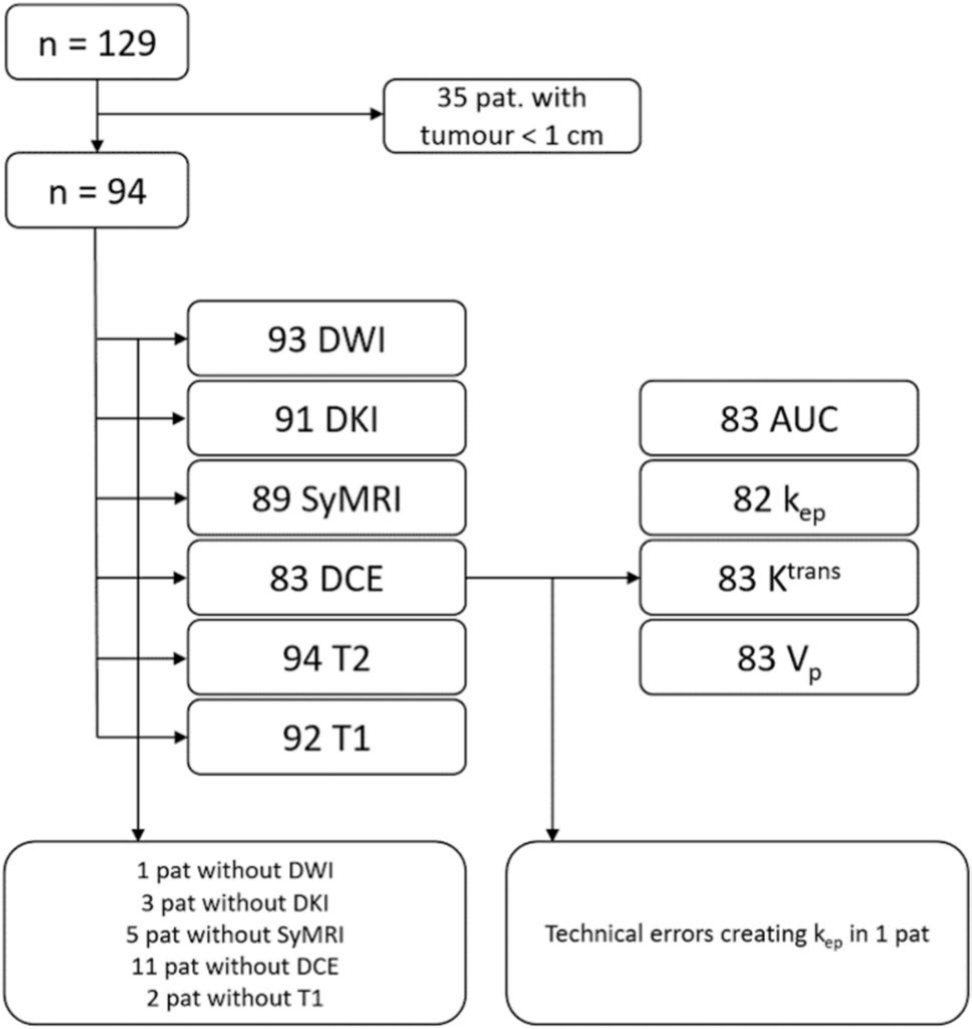
Inclusion chart 129 patients were examined with MRI according to the study protocol. 35 patients had tumours < 1 cm and were excluded, leaving a total of 94 patients. For stated reasons, not all not be calculated. *DKI*   Diffusion kurtosis imaging, *SyMRI*   synthetic MRI, *DWI*   Diffusion weighted imaging, *DCE*   Dynamic contrast enhancement, *k*_*e*p_   reflux time constant, *K*^*trans*^   transfer constant

The total number of included patients was 94 (Fig. 2). Not all sequences were performed in every patient, however 81% of the patients were examined according to the complete MRI protocol. Patient characteristics are described in Table 2.

**Table 2:** Patient and tumour characteristics

Patient characteristics	Value	Range
Included patients	94	
Age median (years) (n = 94)	70	43–88
BMI median (kg/m^2^) (n = 58)	28.7	20–44
FIGO 2009 stage	n	%
I A	50	53.2
I B	19	20.2
II	10	10.6
III A	5	5.3
III B	5	5.3
III C1	4	4.3
III C2	1	1.1
IV	0	0
Tumour type and grade	n	%
Endometroid	79	84
Grade 1	*38/79*	*48.1*
Grade 2	*32/79*	*40.5*
Grade 3	*9/79*	*11.4*
Serous	5	5.3
Carcinosarcoma	4	4.3
Mixed adenocarcionoma	5	5.3
Dedifferentiated carcinoma	1	1.1
Tumour histologic risk group	n	%
Low risk (EC G1-G2)	70	74.5
High risk (EC G3 + non endometroid)	24	25.5

*FIGO*   International Federation of Gynecology and Obstetrics, *EC *  endometrial cancer, *G1*   grade 1, *G2*   grade 2, *G3*   grade 3.

### Association of MRI metrics and tumours of low versus high histological risk

Significant differences were found between patients with low and high histological risk for the mean (p = 0.048), median (*p* = 0.025), skewness (*p* < 0.001) and kurtosis (*p* = 0.003) values derived from the D_app_ series. Significant differences were also found between the groups when comparing kurtosis of v_p_ (*p* = 0.04) and skewness of T2W images (p = 0.037). No significant difference was found between the histological risk groups regarding the quantitative relaxation maps. The main results are presented in Table 3.

**Table 3:** Tumour histogram properties from the different MRI sequences and generated maps

		Mean						Median					
		Histologic risk group						Histologic risk group					
		Low		High		p =	ROC AUC	Low		High		p =	ROC AUC
DWI	ADC	0.76	0.119	0.607	µm^2^/ms	0.131	0.604	0.718	µm^2^/ms	0.697	µm^2^/ms	0.119	0.607
	b1000 map												
	b3500 map												
DKI	D_app_	0.88	***0.025***	0.657	µm^2^/ms	***0.048***	0.639	0.84	µm^2^/ms	0.78	µm^2^/ms	***0.025***	0.657
	K_app_	0.83	0.381	0.561		0.59	0.538	0.857		0.882		0.381	0.561
Synthetic MRI	PD	80.7	0.308	0.578	%	0.275	0.581	80.7	%	81.2	%	0.308	0.578
	T1	1498	0.917	0.507	ms	0.887	0.510	1398	ms	1402	ms	0.917	0.507
	T2	79.8	0.129	0.608	ms	0.114	0.613	79	ms	83.7	ms	0.129	0.608
Perfusion	AUC	327	0.884	0.508	mM*s	0.669	0.517	229	mM*s	225	mM*s	0.884	0.508
	k_ep_	0.118	0.56	0.539	min^−1^	0.269	0.583	0.089	min^−1^	0.096	min^−1^	0.56	0.539
	K^trans^	0.161	0.558	0.544	min^−1^	0.741	0.525	0.08	min^−1^	0.099	min^−1^	0.558	0.544
	v_p_	235	0.075	0.633		0.054	0.644	121		65		0.075	0.633
T1W MRI	T1W												
	T1W fat sat												
T2W MRI	T2W												

Skewness				Kurtosis			
Histologic risk group				Histologic risk group			
Low	High	p =	ROC AUC	Low	High	p =	ROC AUC
0.683	0.854	0.588	0.516	4.02	4.11	0.269	0.530
− 0.297	− 0.214	0.954	0.521	2.83	3.30	0.182	0.592
− 0.121	− 0.241	0.216	0.595	2.88	3.07	0.322	0.546
0.825	1.44	** < *0.001***	***0.744***	3.75	5.80	***0.003***	0.708
− 0.621	− 0.721	0.971	0.503	5.08	4.37	0.609	0.536
0.03	0.045	0.869	0.514	3.23	3.19	0.242	0.583
1.32	1.31	0.976	0.542	5.48	5.64	0.842	0.514
0.706	0.701	0.549	0.543	4.31	4.30	0.725	0.525
3.59	3.51	0.558	0.544	21.9	20.5	0.53	0.547
0.821	0.831	1	0.500	3.11	3.61	0.674	0.531
3.9	3.08	0.609	0.538	20.1	17.0	0.924	0.507
3.55	4.39	0.058	0.641	18.1	33.9	***0.04***	0.653
− 0.022	0.021	0.887	0.510	3.24	3.45	0.965	0.503
− 0.001	0.133	0.294	0.572	3.28	3.45	0.332	0.567
0.39	0.102	***0.037***	0.643	3.68	3.26	0.19	0.590

This table shows the histogram properties from the segmented parts of the tumours on all available MRI maps. The values were compared with either t-test or non-parametric test depending on distribution. The diagnostic performance in classifying the tumour as low or high histologic risk was analysed with an ROC analysis, presented here as the area under curve (ROC AUC) Significant differences and the highest ROC AUC are marked in bold italic letters

*DWI*   diffusion weighted imaging, *ADC*   apparent diffusion coefficient, *DKI*   diffusion kurtosis imaging, *D*_*app*_   diffusion coefficient corrected to account for the observed non-Gaussian behaviour, *K*_*app*_   apparent kurtosis coefficient, *AUC*   area under curve, *k*_*ep*_   exchange rate constant, *K*^*trans*^   transfer coefficient, *v*_*p*_   plasma volume fraction, *PD*   proton density, *ROC*   Receiver operating characteristic

**Fig. 3:**
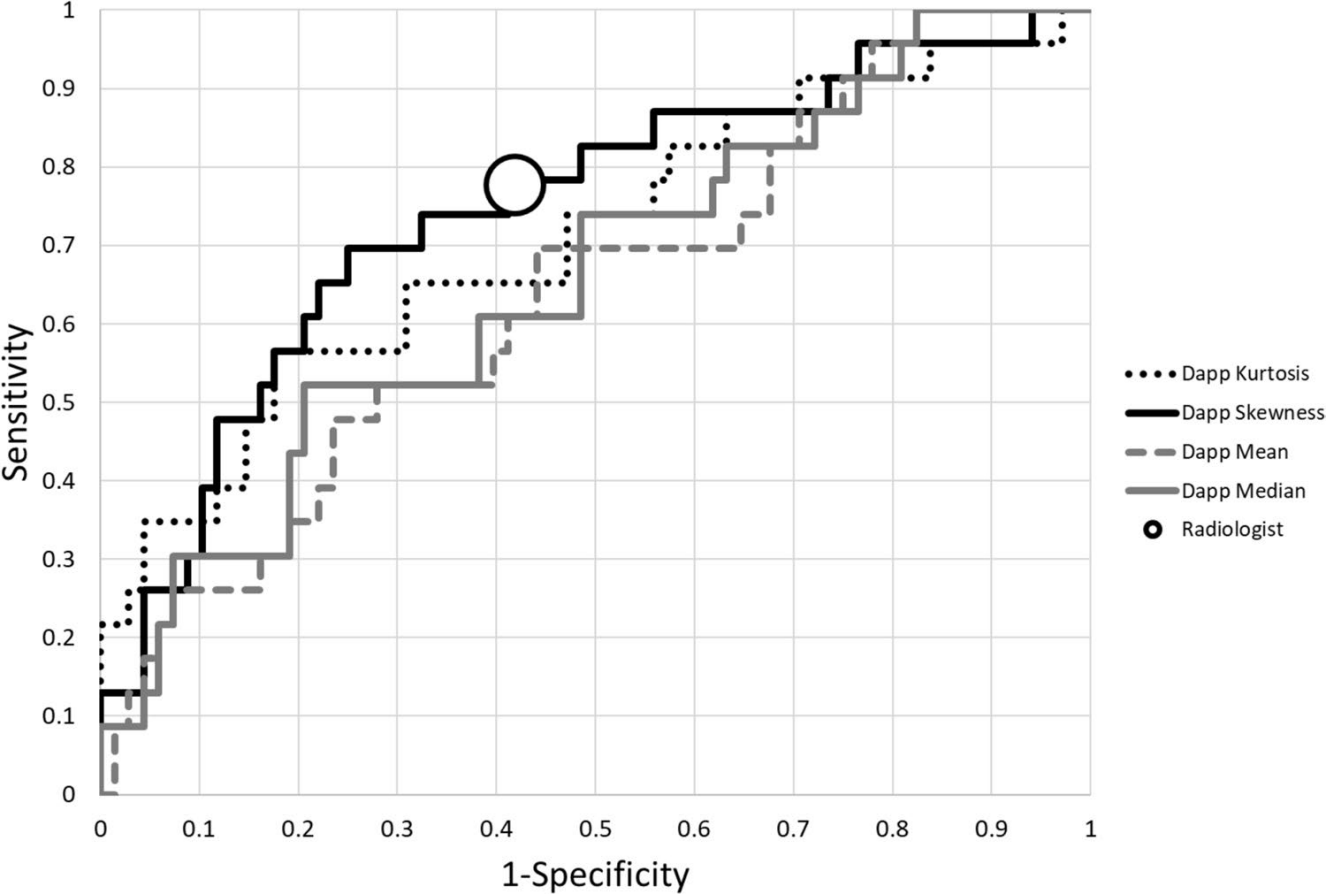
ROC analysis of the diagnostic performances of histogram properties from D_app_ in discriminating between low and high risk histologic EC. Radiologist performance: sensitivity = 0.778, specificity = 0.582. D_app_ = diffusion coefficient corrected to account for the observed non-Gaussian behaviour

An ROC analysis of all included histogram properties (Table 3) calculating the AUC showed the highest area (0.744) for the histogram skewness of D_app_ followed by the kurtosis of the D_app_ map histograms (0.708). ROC curves of the D_app_ histogram properties is shown in Fig. 3.

### Association of MRI metrics and FIGO grades

**Fig. 4:**
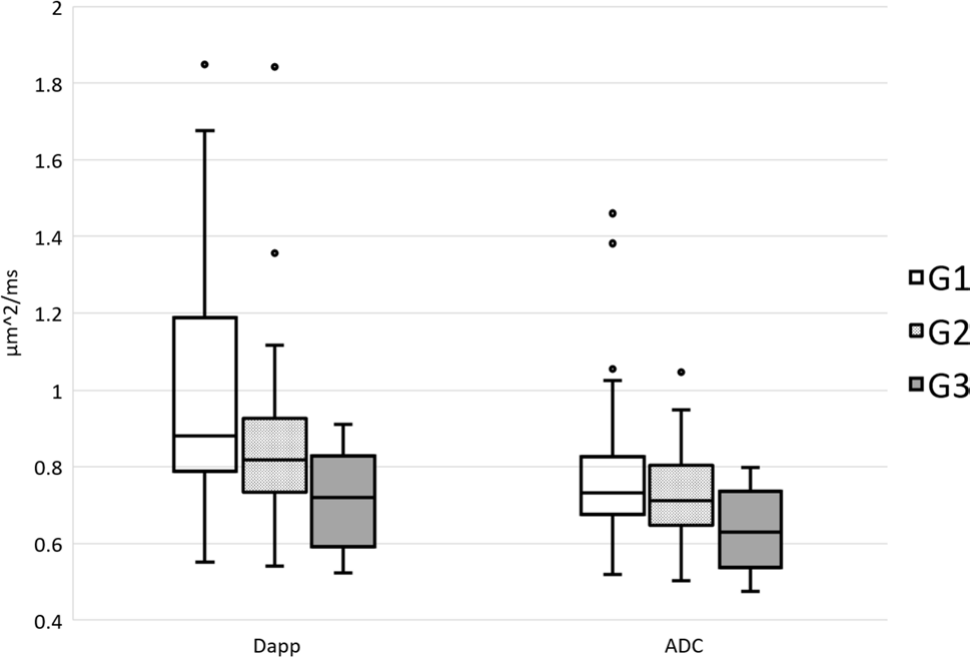
Median D_app_ and ADC values (µm^2^/ms) of EC histologic FIGO grades (G1 = FIGO grade 1, G2 = FIGO grade 2, G3 = FIGO grade 3) D_app_ = diffusion coefficient corrected to account for the observed non-Gaussian behaviour, ADC = apparent diffusion coefficient, EC = endometrial cancer, FIGO = International Federation of Gynecology and Obstetrics.

There were significant differences between the histogram properties of D_app_ among the different FIGO grades of EC, especially regarding skewness and kurtosis (p < 0.001), but also in mean and median values as shown in Table 4. The differences in histogram properties of ADC were not as pronounced. Figure 4 illustrates differences in median values.

**Table 4:** ANOVA analysis of histogram properties from D_app_ and ADC in the different FIGO-grades of endometroid cancer

Histogram property		FIGO grade 1		FIGO grade 2		FIGO grade 3		
		n = 37		n = 31		n = 9		
		Mean	SD	Mean	SD	Mean	SD	*p* =
ADC	Kurtosis	4.735	0.339	4.622	0.283	5.507	1.119	0.499
	Mean (µm^2^/ms)	0.821	0.037	0.754	0.026	0.658	0.041	0.052
	Median (µm^2^/ms)	0.803	0.037	0.735	0.023	0.640	0.038	***0.041***
	Skewness	0.704	0.132	0.630	0.137	0.616	0.367	0.924
D_app_	Kurtosis	3.801	0.293	4.809	0.371	9.300	2.055	** < *0.001***
	Mean (µm^2^/ms)	1.019	0.052	0.908	0.049	0.751	0.046	***0.032***
	Median (µm^2^/ms)	0.990	0.053	0.862	0.044	0.704	0.045	***0.014***
	Skewness	0.682	0.101	0.993	0.097	1.719	0.293	** < *0.001***

Significant differences in histogram properties from MRI images of tumours with different FIGO grades marked in bold italic letters

*FIGO*   International Federation of Gynecology and Obstetrics, *ADC*   apparent diffusion coefficient, *D*_*app*_ Diffusion coefficient corrected to account for the observed non-Gaussian behaviour

### Radiological assessment

The radiologist had a sensitivity of 77.8%, a specificity of 58.2%, a 20.0% positive predictive value and a 95.1% negative predictive value in discriminating histologic high-risk tumours from histologic low-risk tumours.

### Discussion

This prospective study investigated differences in tumour histogram properties of images from conventional MRI as well as of images from sequences not in clinical use between EC of low or high histological risk. The main result indicates that histogram features of D_app_ maps from diffusion kurtosis imaging may be able to preoperatively discriminate between EC of high and low histological risk.

Some previous studies have reported lower ADC-values in G3 tumours than in G1 and G2 [16, 17, 36], while other studies reported that ADC was not useful to grade the tumour [18, 37–39].

**Fig. 5:**
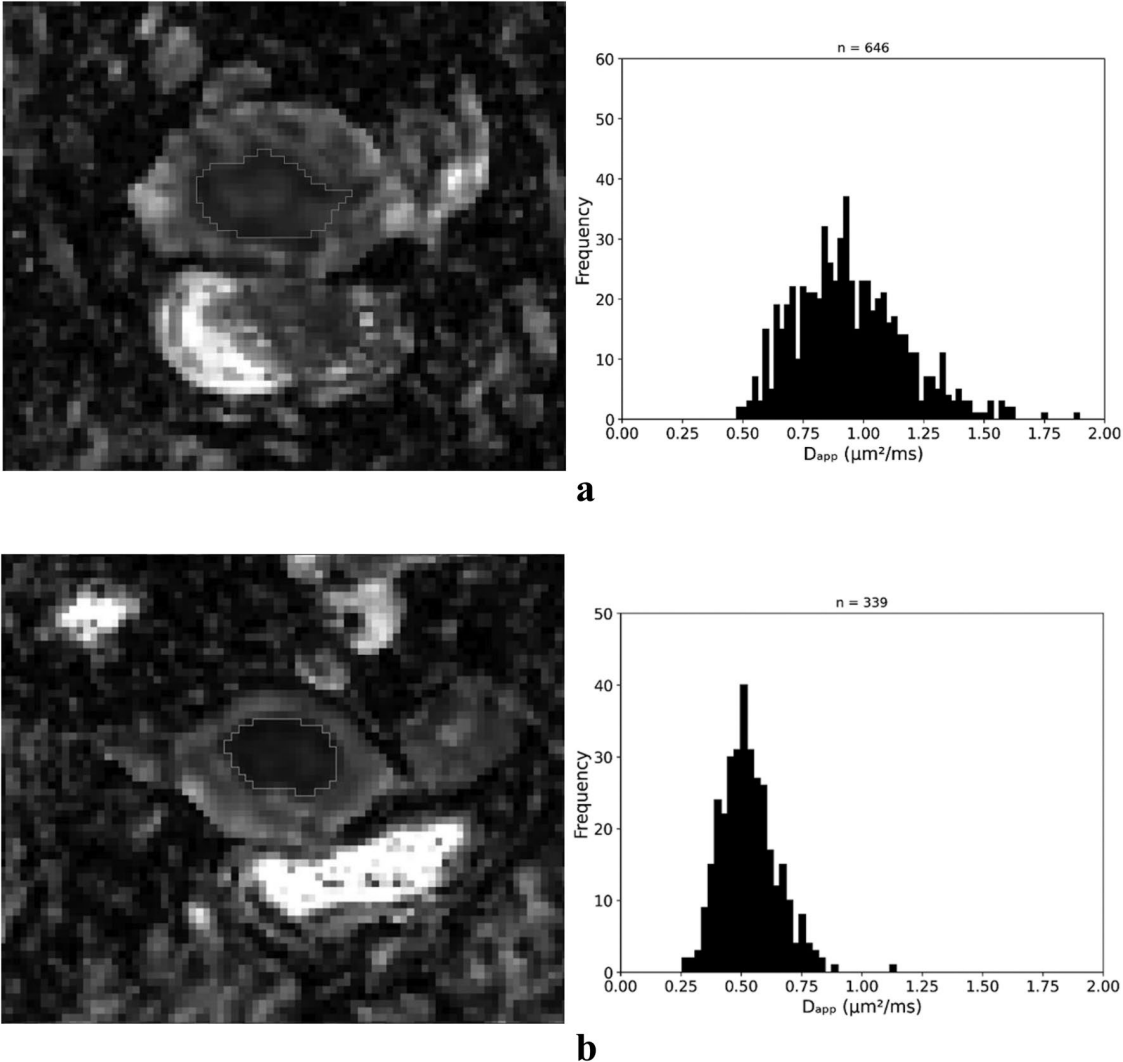
D_app_ MR images and histograms of one tumour with low histologic risk (**a**) and one with high histologic risk (**b**). Higher kurtosis is seen for the tumour with high risk histology (n = number of pixels). D_app_ = diffusion coefficient corrected to account for the observed non-Gaussian behaviour

The differences in D_app_ may better reflect the more heterogeneous cellularity of more aggressive tumours. This has previously been shown in other tumours such as gliomas, prostate cancer, and breast cancer [40–42]. In EC, D_app_ seems to correlate with tumour grade and may be better at discriminating tumour grades or histological risk groups than ADC in retrospective studies [43, 44]. However, one prospective study showed that while DKI could discriminate between histological risk groups, it did not perform significantly better than ADC [45]. In our prospective study, there was a statistically significant difference in D_app_ but not in ADC in tumours of different histological risk. Our results agree with previous studies regarding D_app_ despite using different b values for the calculations. Further, our results indicate that DKI may better correlate with tumour grade than ADC, in agreement with most previous studies on EC [27, 43–45]. Figure 5 illustrates D_app_ MR images and histograms from two tumours from different histological risk groups. The lower D_app_ mean and median values in the higher grades of EC may be due to the higher proportion of solid tumour component in the higher grade tumours. These findings should next be validated on different MRI scanners and in other hospitals to determine their generalizability as a step towards clinical applicability.

Future studies may explore the possibilities of other radiomic features of D_app_ of EC tumours such as the 10th and 90th percentile, the entropy, or the spatial arrangement of voxel intensities as well as including DKI in more general predictive models including clinical data and comparing other groups than histological tumour risk.

One previous study examined EC using relaxation maps acquired by synthetic MRI where a difference in relaxation times and proton density between tumours with and without deep myometrial invasion was identified, but no significant difference when comparing tumour grades [15]. However, they compared G1 to G2-G3, not low- and high-risk histology as in the present study. The discriminating capability regarding myometrial invasion was not investigated in this study. Our results are in line with theirs regarding tumour grade, indicating that quantitative relaxation times do not aid the preoperative diagnostics.

The differences in vascularization detected by TVUS described by Epstein et al. [14] were not corroborated using DCE and histogram analysis of the perfusion maps. Previous studies on DCE MRI perfusion in EC showed association between low K^trans^ and high-risk tumours/poorer prognosis [28–31]. In our study, the largest in comparison (n = 83 patients versus n = 44–74), no such association was found. The significant difference in the kurtosis of the histograms from v_p_ maps, depicting the plasma volume, may indicate heterogeneity in the microvasculature. Ideally, we would have analysed histograms from maps of extravascular extracellular space (v_e_) as well. Our measurement results of the parameter were implausible, however, and we decided to omit it from the analysis after consultation with the scanner manufacturer.

The diagnostic performance when analysing the skewness of histograms extracted from DKI derived D_app_ maps is comparable to the diagnostic performance of a radiologist with more than 20 years of experience in gynaecological MRI, indicating the possible potential of quantitative analysis.

If these results are validated, a possible integration into clinical practice could be to either confirm or question the histopathologic report from the preoperative biopsy. This may have the potential to reduce the percentage of tumours being wrongly classified preoperatively and thereby reducing the number of both unnecessary extensive surgery and the need of a second surgical procedure.

A strength of this study is the prospective design, where all included patients were examined according to the same protocol and on the same MRI scanner, avoiding inconsistencies between scanners and protocols. Another strength is the extended protocol used, including quantitative T1W and T2W maps acquired by synthetic MRI, and DWI with extra high b-values, allowing us to compare the histogram properties of different sequences on the same tumours. Another strength is that the risk-group stratification was based on reference histopathology on surgical specimens for all participants.

A limitation of this study is the relatively small number of included tumours with high histological risk. However, we included a few more patients than the comparable study of DKI [45] and only slightly fewer than the comparable prospective study on synthetic MRI [15].

Another limitation may be that we segmented tumours only on three selected adjacent slices, rather than on all slices where the tumour was visible. However, since macro-morphologically, EC is a relatively homogenous tumour, there is reason to believe that our results are comparable to whole-tumour segmentations. Whole tumour segmenting would have included a higher number of pixels in the histograms, making the extracted properties more robust. There is also a risk that important parts of the tumours were missed using our approach. Given the time savings achieved through slice selection though, our approach might even be considered a strength. However, automated segmentation may solve this issue in the future [46].

A further limitation may be that the number of segmented pixels differed between the MRI sequences, related to the different spatial resolution of the images. When comparing ADC to D_app_, the histograms for the latter were based on fewer pixels.

## Conclusion

DKI has the potential to better reflect differences in tumour histology compared to conventional DWI and may be useful in preoperative risk stratification of EC, pending further validation.

## Data Availability

The data that support the findings of this study are not openly available due to reasons of sensitivity and are available from the corresponding author upon reasonable request.

## Abbreviations

ADC: Apparent diffusion coefficient
AUC: Area under curve
D_app_: Diffusion coefficient corrected to account for the observed non-Gaussian behaviour
DKI: Diffusion kurtosis imaging
DWI: Diffusion weighted imaging
EC: Endometrial cancer
EES: Extravascular extracellular space
ESUR: European Society of Urogenital Radiology
FIGO: FIGO tumour grade 1, 2 and 3
G1, G2, G3: Apparent diffusion coefficient
Gd: Gadolinium
K_app_: Apparent diffusional kurtosis
k_ep_: Time constant for reflux of gadolinium from the extravascular extracellular space
K^trans^: Transfer constant for gadolinium form plasma to extravascular extracellular space
v_p_: Fractional plasma volume contributing to the intravascular part of the signal
