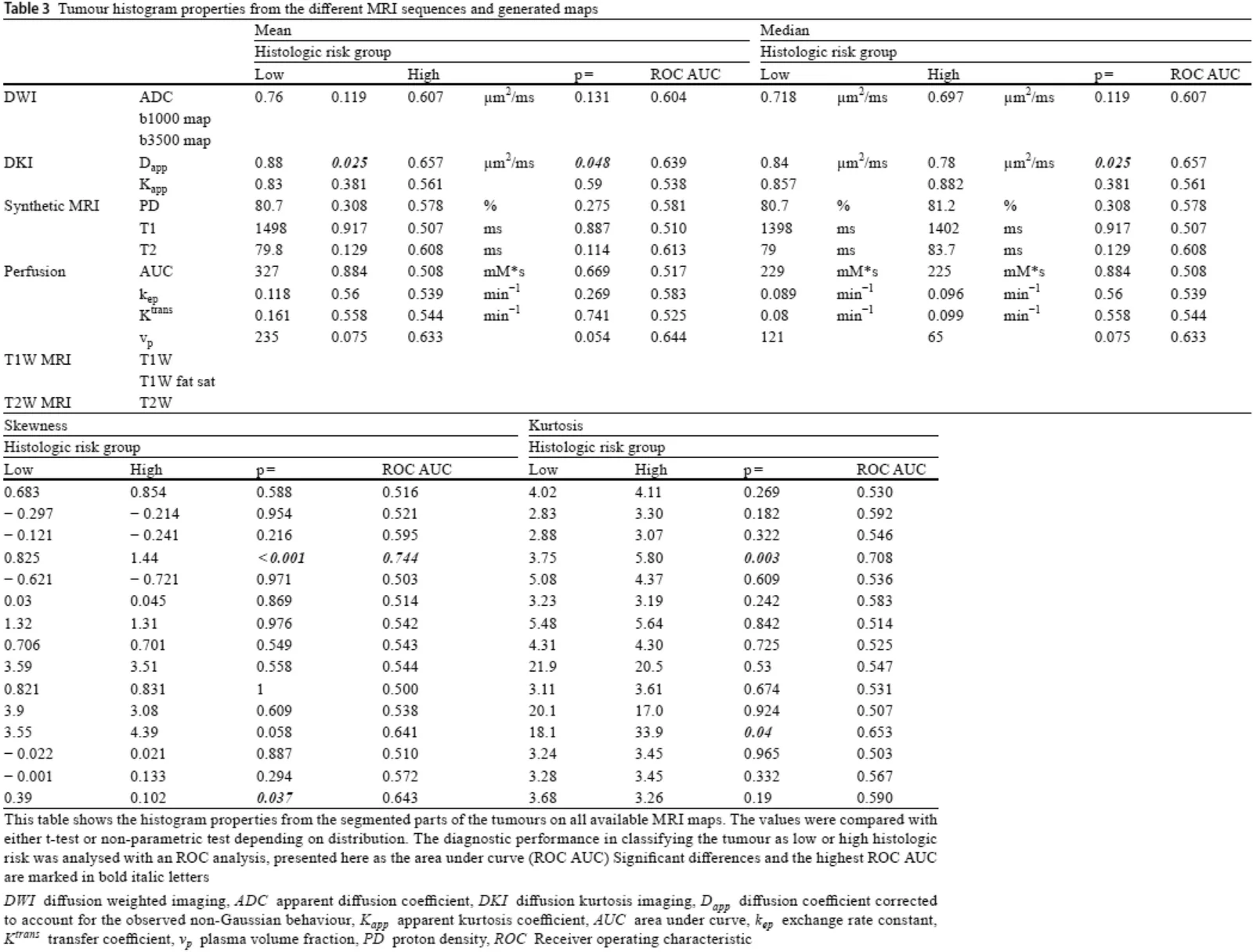# Correction to: Preoperative risk assessment of endometrial cancer using histogram analysis of weighted and quantitative MRI images

**DOI:** 10.1007/s00261-026-05489-y

**Published:** 2026-05-21

**Authors:** Magnus Palmér, Åsa Åkesson, Maria Ljungberg, Stefan Kuczera, Emilia Gryska, Erica de Coursey, Rolf A. Heckemann, Pernilla Dahm Kähler, Stephan E. Maier, Henrik Leonhardt

**Affiliations:** 1 University of Gothenburg, Gothenburg, Sweden; 2 Sahlgrenska University Hospital, Gothenburg, Sweden; 3 Brigham and Women’s Hospital, Boston, United States

**Correction to: Abdominal Radiology (2026) 51:346–356**

https://doi.org/10.1007/s00261-025-05069-6

In the original article, in Table 3, the mean values in the high histologic risk group are incorrect. The old incorrect version and the corrected version of Table 3 are displayed below.

 In addition, the surname of co-author name “Stefan Kuzcera” is incorrect. The author name should read as “Stefan Kuczera” instead “Stefan Kuzcera”. The original article has been corrected.

Correct version of Table 3:

**Table 3:** Tumour histogram properties from the different MRI sequences and generated maps

		Mean						Median					
		Histologic risk group						Histologic risk group					
		Low		High		p=	ROC AUC	Low		High		p=	ROC AUC
DWI	*ADC*	0.76	µm^2^/ms	0.72	µm^2^/ms	0.131	0.604	0.718	µm^2^/ms	0.697	µm^2^/ms	0.119	0.607
	*b1000 map*												
	*b3500 map*												
DKI	*D*_*app*_	0.88	µm^2^/ms	0.83	µm^2^/ms	***0.048***	0.639	0.84	µm^2^/ms	0.78	µm^2^/ms	***0.025***	0.657
	*K*_*app*_	0.83		0.84		0.59	0.538	0.857		0.882		0.381	0.561
Synthetic MRI	*PD*	80.7	%	81.3	%	0.275	0.581	80.7	%	81.2	%	0.308	0.578
	*T1*	1498	ms	1503	ms	0.887	0.510	1398	ms	1402	ms	0.917	0.507
	*T2*	79.8	ms	85	ms	0.114	0.613	79	ms	83.7	ms	0.129	0.608
Perfusion	*AUC*	327	mM*s	313	mM*s	0.669	0.517	229	mM*s	225	mM*s	0.884	0.508
	*k*_*ep*_	0.118	min^−1^	0.112	min^−1^	0.269	0.583	0.089	min^−1^	0.096	min^−1^	0.56	0.539
	*K* ^*trans*^	0.161	min^−1^	0.166	min^−1^	0.741	0.525	0.08	min^−1^	0.099	min^−1^	0.558	0.544
	*v*_*p*_	235		125		0.054	0.644	121		65		0.075	0.633
T1W MRI	*T1W*												
	*T1W fat sat*												
T2W MRI	*T2W*												

		Skewness				Kurtosis			
		Histologic risk group				Histologic risk group			
		Low	High	p=	ROC AUC	Low	High	p=	ROC AUC
DWI	*ADC*	0.683	0.854	0.588	0.516	4.02	4.11	0.269	0.530
	*b1000 map*	−0.297	−0.214	0.954	0.521	2.83	3.30	0.182	0.592
	*b3500 map*	−0.121	−0.241	0.216	0.595	2.88	3.07	0.322	0.546
DKI	*D*_*app*_	0.825	1.44	***<0.001***	***0.744***	3.75	5.80	***0.003***	0.708
	*K*_*app*_	−0.621	−0.721	0.971	0.503	5.08	4.37	0.609	0.536
Synthetic MRI	*PD*	0.03	0.045	0.869	0.514	3.23	3.19	0.242	0.583
	*T1*	1.32	1.31	0.976	0.542	5.48	5.64	0.842	0.514
	*T2*	0.706	0.701	0.549	0.543	4.31	4.30	0.725	0.525
Perfusion	*AUC*	3.59	3.51	0.558	0.544	21.9	20.5	0.53	0.547
	*k*_*ep*_	0.821	0.831	1	0.500	3.11	3.61	0.674	0.531
	*K* ^*trans*^	3.9	3.08	0.609	0.538	20.1	17.0	0.924	0.507
	*v*_*p*_	3.55	4.39	0.058	0.641	18.1	33.9	***0.04***	0.653
T1W MRI	*T1W*	−0.022	0.021	0.887	0.510	3.24	3.45	0.965	0.503
	*T1W fat sat*	−0.001	0.133	0.294	0.572	3.28	3.45	0.332	0.567
T2W MRI	*T2W*	0.39	0.102	***0.037***	0.643	3.68	3.26	0.19	0.590

This table shows the histogram properties from the segmented parts of the tumours on all available MRI maps. The values were compared with either t-test or non-parametric test depending on distribution. The diagnostic performance in classifying the tumour as low or high histologic risk was analysed with an ROC analysis, presented here as the area under curve (ROC AUC) Significant differences and the highest ROC AUC are marked in bold italic letters

*DWI*   diffusion weighted imaging, *ADC*   apparent diffusion coefficient, *DKI*   diffusion kurtosis imaging, *D*_*app*_   diffusion coefficient corrected to account for the observed non-Gaussian behaviour, *K*_*app*_   apparent kurtosis coefficient, *AUC*   area under curve, *k*_*ep*_   exchange rate constant, *K*^*trans*^   transfer coefficient, *v*_*p*_   plasma volume fraction, *PD*   proton density, *ROC*   Receiver operating characteristic

Incorrect version:

**Figure:**